# Genome-Wide Identification and Expression Analysis of Chitinase Genes in Watermelon under Abiotic Stimuli and *Fusarium oxysporum* Infection

**DOI:** 10.3390/ijms25010638

**Published:** 2024-01-04

**Authors:** Changqing Xuan, Mengjiao Feng, Xin Li, Yinjie Hou, Chunhua Wei, Xian Zhang

**Affiliations:** 1 State Key Laboratory of Crop Stress Biology in Arid Areas, College of Horticulture, Northwest A & F University, Xianyang 712100, China; xuanchangqing@nwafu.edu.cn (C.X.); fmj@nwafu.edu.cn (M.F.); xinli258@nwafu.edu.cn (X.L.); hyj1224@nwafu.edu.cn (Y.H.); 2 State Key Laboratory of Vegetable Germplasm Innovation, Tianjin 300384, China

**Keywords:** chitinase, watermelon, genome-wide identification, expression analyses, abiotic stresses, *Fusarium oxysporum*

## Abstract

Chitinases, which catalyze the hydrolysis of chitin, the primary components of fungal cell walls, play key roles in defense responses, symbiotic associations, plant growth, and stress tolerance. In this study, 23 chitinase genes were identified in watermelon (*Citrullus lanatus* [Thunb.]) and classified into five classes through homology search and phylogenetic analysis. The genes with similar exon-intron structures and conserved domains were clustered into the same class. The putative *cis*-elements involved in the responses to phytohormone, stress, and plant development were identified in their promoter regions. A tissue-specific expression analysis showed that the *ClChi* genes were primarily expressed in the roots (52.17%), leaves (26.09%), and flowers (34.78%). Moreover, qRT-PCR results indicate that ClChis play multifaceted roles in the interaction between plant/environment. More *ClChi* members were induced by Race 2 of *Fusarium oxysporum* f. sp. *niveum*, and eight genes were expressed at higher levels on the seventh day after inoculation with Races 1 and 2, suggesting that these genes play a key role in the resistance of watermelon to Fusarium wilt. Collectively, these results improve knowledge of the chitinase gene family in watermelon species and help to elucidate the roles played by chitinases in the responses of watermelon to various stresses.

## 1. Introduction

Chitin, as the second most abundant polysaccharide polymer in nature, is formed by β-1,4-linked N-acetyl-D-glucosamine (GlcNAc) and is found widely in crustacean shells, insect and arthropod exoskeletons, nematode cuticles, and the cell walls of fungi and diatoms [1,2,3]. Chitinases are one group of glycosyl hydrolases (GH), which can hydrolyze chitin into *N*-acetylglucosamines. In addition, chitinases can be divided into endochitinases (EC 2.2.1.14) and exochitinases (EC 3.2.1.52), depending on the location of action and the end product. The former randomly hydrolyzes chitin to produce oligomers, which release different sizes of mixed end products, while they can cleave chitin and chitin oligomers from their non-reducing end [4]. On the other side, according to the sequence homology of catalytic domains of plant chitinases, they are placed in two major glycosyl hydrolase families, 18 and 19 (GH18 and GH19, respectively) [5]. Furthermore, based on the relationships of structure, evolution, catalytic reaction, function, and substrates of chitinases, they are classified into seven distinct classes (Class I–VII) [6,7,8,9]. GH18 contains Class III and Class V chitinases, and GH19 includes classes I, II, IV, VI, and VII [10]. In addition, in contrast to the GH19 chitinases, which are virtually exclusively found in higher plants, those of the GH18 family are extensively dispersed among organisms [11].

The ability to degrade chitin is found in the species that contain chitin, such as insects, crustaceans, and fungi, among others. It is necessary for the deacetylation of chitin to change the physical and chemical properties of the cuticles, cytoderms, and shells, which make them soft and soluble in insects, fungi, and crustaceans, respectively [6,12]. However, the chitinases occur widely in higher plants, including monocots and dicots, which do not contain chitin. To date, chitinases have been found in the *Arabidopsis thaliana* (hereafter *Arabidopsis*) and rice [13], maize [14], sorghum [15], soybean [16], black bean [17], barley [18], cabbage [19], banana [20], cucumber [6], garlic [21], tea [22], muskmelon [23], tomato [24], mulberry [25] and wheat [26]. Chitinases are considered to belong to the category of pathogenesis-related (PR) proteins. Class I and Class II chitinases are included in PR-4; the chitinases in classes I, II, IV, VI, and VII are included in PR-3; and Class III and Class V chitinases are included in PR-8 and PR-11, respectively [27,28]. Chitinases play a defense role by hydrolyzing the major structural component of insects and fungi and are induced indirectly by the activation of systemic acquired resistance (SAR) or the hypersensitive response (HR) in plants [29]. Moreover, chitinases are involved in the effector-triggered immunity (ETI) pathway mediated by salicylic acid (SA) or the HR induced in plants [30], and plant pathogens-associated molecular pattern (PAMP) -triggered immunity (PTI), owing to the fact that the substrate chitin is well known as a pathogens-associated molecular pattern PAMP. For example, the plantlets of tea overexpressed a Class I chitinase gene from potato that harbored a resistant blister blight disease phenotype by forming HR in the inoculated area [31]. A lysozyme-like hydrolase (hydrolase 1, LYS1), which is a type of chitinase, was produced from a bacterial infection in *Arabidopsis*, and the knockdown *LYS1* mutant was hyper susceptible to bacterial infection [32]. In addition, the heterologous expression of *MnChi18* (from mulberry) in *Arabidopsis* significantly enhanced the amount of resistance to gray mold (*Botrytis cinerea*), increased the activity of catalase, and decreased the content of malondialdehyde (MDA) in the overexpressed plants [25]. Moreover, the genes related to resistance, such as β-1,3-glucanase 2 (*BG2*) and hypersensitive-induced reaction 1 (*HIR1*), were significantly upregulated in the transgenic plants. The growth of the fungus and the development of leaf necrosis was inhibited in the transgenic cacao plants that endogenously overexpressed the Class I chitinase gene *TcChi1* compared to the control [33]. In addition, examples of the endogenous or heterologous expression of chitinase genes to improve plant resistance to fungi or bacteria have also been found in tobacco [34], poplars [35], maize [36], tea [31], oriental melon [37], and other plants.

Chitinases are not only involved in the response to biotic stresses but also to abiotic stresses, including drought, high salinity, cold, wounding, heavy metal pollution, and ultraviolet light [38]. In pepper plants, the knockdown of chitinase gene *CaChiIV1* increased its sensitivity to infection by the oomycete *Phytophthora capsici*, and the root activity decreased following treatment with mannitol [39]. Moreover, the relative excessive electrolyte leakage and significant reduction in total chlorophyll in the leaves of *CaChiIV1*-silenced plants treated with mannitol revealed that *CaChiIV1* has a major function in the response to drought stress. *LcCHI2* that encodes a Class II chitinase from *Leymus chinensis* was overexpressed in tobacco and maize, and the transgenic plants accumulated low levels of Na^+^ and MDA and had a reduction in their relative electrical conductivity under salt stress [40]. In addition, the chitinase genes, *CpCHT1*, *BiCHT1*, *CHT9,* and *CHT46,* were isolated from wintersweet, bromegrass, and rye, which encode antifreeze proteins to protect these plants from freezing injury [41,42,43]. The overexpression of *CHIT33* and *CHIT42* from *Trichoderma harzianum* conferred resistance to heavy metals, such as copper (Cu), mercury (Hg), and cadmium (Cd), in tobacco plants [44]. Different chitinase isoforms were induced by lead (Pb), Cd, and arsenic (As) in faba bean, soybean, pea, barley, dwarf sunflower, and maize [45,46]. In addition, SA, ultraviolet C light, and wounding trigger the accumulation of *IF3* mRNA and the protein it encodes in lupin [47].

Chemical compounds, such as abscisic acid (ABA), jasmonic acid (JA), salicylic acid (SA), ethylene (ETH), systemin, and bioelectrical and hydraulic signals, are considered to be wounding or defense-related signals in plants [48,49,50]. Consistent with their role in plant defense, chitinase genes are often induced by these phytohormones in response to wounding or environmental stress. The level of expression of *AtChiC* in *Arabidopsis* was significantly induced by ABA and JA, which are plant hormones related to stress [51]. Moreover, the strawberry chitinase gene *FnCHIT2* was highly induced by SA, and the ectopic expression of *FnCHIT2* in *Arabidopsis* enhanced its resistance to *Colletotrichum higginsianum* and *Pseudomonas syringae* pv. tomato DC3000 [52]. In kiwifruit, treatment with methyl jasmonate (MeJA) enhanced the activity of *AcCHI* and alleviated the damage from *Botryosphaeria dothidea* [53]. Overexpression of the cotton chitinase gene *GhChi6* in *Arabidopsis* plants improved the levels of transcripts of the key genes involved in the SA signaling pathway that improved the resistance of transgenic *Arabidopsis* plants to aphids, while the expression levels of the genes involved in the JA and ETH signaling pathways were reduced compared to the wild-type plants [54].

Watermelon is a strong economic value crop that is in the Cucurbitaceae family. During its growth and developmental process, the plants often suffer from biological and abiotic stress, such as drought, low temperature, salinity, wilt, and powdery mildew. In previous studies, chitinase was considered to belong to a class of PR proteins which play important roles in the interaction between plants, microorganisms, and stress and the regulation of plant growth and development in many species. However, their potential functions have not been studied in watermelon. In this study, 23 chitinase genes were identified in watermelon and designated *ClChi* genes. Moreover, their distribution on chromosomes, structure, evolutionary relationships, and patterns of expression in response to plant hormones and biological/abiotic stress were further analyzed. This study will help to dissect the important roles of *ClChit* genes involved in the growth and development of watermelon and its resistance to external stress factors.

## 2. Results

### 2.1. Genome-Wide Identification and Synteny Analysis of the ClChi Genes

**Figure 1:**
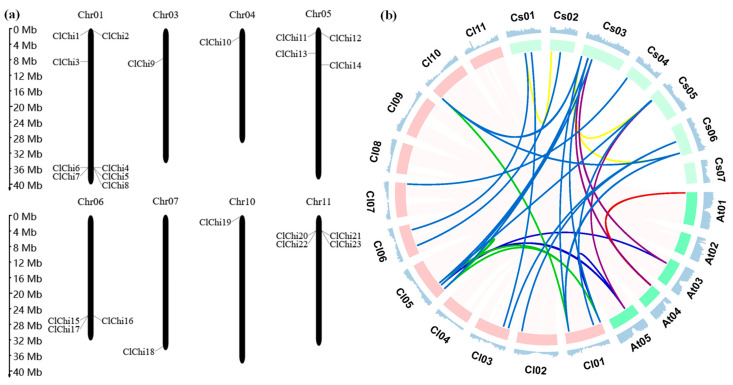
(**a**) Distribution of 23 *ClChi* genes on the watermelon chromosomes. The scale ruler on the left side shows the physical distance (Mb) of the chromosomes. The relative positions of the *ClChis* are marked on the chromosomes. (**b**) Syntenic relationships among the *Arabidopsis*, cucumber, and watermelon chitinase genes are indicated in different colors; Red: *Arabidopsis* vs. *Arabidopsis*; Green: watermelon vs. watermelon; Yellow: cucumber vs. cucumber; Blue: watermelon vs. cucumber; Dark blue: *Arabidopsis* vs. watermelon; Purple: *Arabidopsis* vs. cucumber.

In this study, 23 putative chitinase genes were identified in watermelon by exploring the whole-genome sequences of *Citrullus lanatus subsp. vulgaris cv.* 97103 (ver. 2.5) and the homologous sequences of chitinase in *Arabidopsis* as queries. A chromosomal distribution map was constructed from watermelon based on the position of each chitinase gene, and these putative chitinase genes were designated from *ClChi1* to *ClChi 23* (Figure 1a). All the predicted chitinases were physically mapped to the watermelon chromosomes, except for chromosome numbers 2, 8, and 9. Eight *ClChi* genes were contained in chromosome 1, whereas chromosomes 3, 4, 7, and 10 each contained just one *ClChi* gene. In addition, chromosomes 5 and 11 each contained four *ClChi* genes, and chromosome 6 contained three *ClChi* genes. Table 1 shows detailed information about the predicted *ClChi* genes, and their coding domain sequences ranged from 828 (ClChi9) to 2061 bp (ClChi18). In addition, they encoded putative peptides that range from 276 to 687 aa, and their molecular weights ranged from 29.47 to 75.71 kDa. The theoretical isoelectric points (pI) ranged from 4.46 (ClChi2) to 9.81 (ClChi14). An analysis to predict the signal peptides showed that 16 of the 23 ClChis contained signal peptides, and the subcellular localization prediction indicated that the ClChi members have extracellular, cytoplasmic, chloroplastic, vacuolar, and nuclear functions.

Since the duplication of genes in different plants is important to format the homologous genes and study their evolution, the replication of chitinase genes in watermelon and the synteny relationships between watermelon, cucumber, and *Arabidopsis* of the chitinase genes were analyzed (Figure 1b). In the watermelon genome, there were 13 and 3 *ClChi* gene members that contained 1–2 copies, and 1–3 copies were found in *Arabidopsis* and cucumber, respectively (Table S1). However, only three *CsChi* genes contained just one copy in *Arabidopsis*. Among the chromosomes of *Arabidopsis*, cucumber, and watermelon, one pair of *AtChi*, three pairs of *CsChi*, and four pairs of *ClChi* were found to be segmentally duplicated.

### 2.2. Phylogenetic and Conserved Domain Analysis of the ClChi Genes

**Figure 2:**
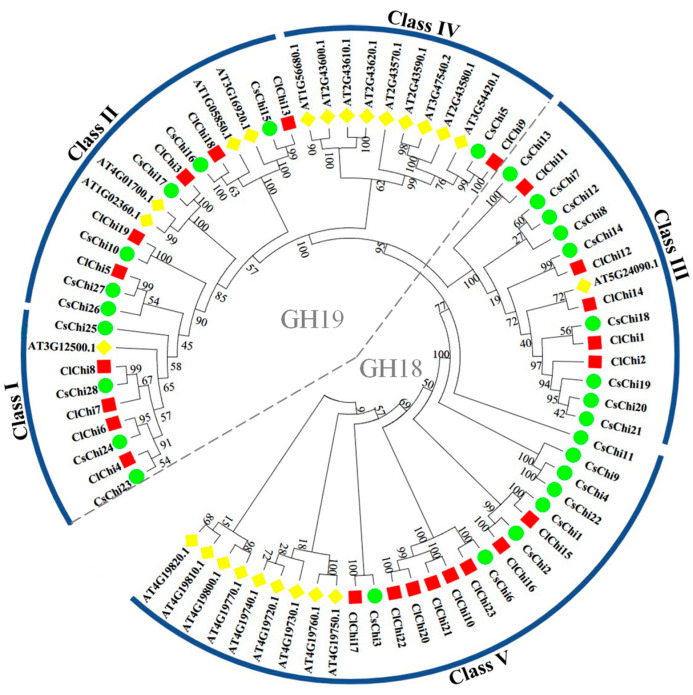
The unrooted phylogenetic tree of chitinase genes was generated based on the amino acid sequences of *Arabidopsis*, cucumber, and watermelon by the neighbor-joining (NJ) method using MEGA 7.0. The chitinase members were categorized into five clades and labeled as I, II, III, IV, and V. The chitinase members of species were color-coded: At, *Arabidopsis* (yellow); Cs, cucumber (green); Cl, watermelon (red).

The neighbor-joining (NJ) method was used to build an unrooted phylogenetic tree that included the 24, 28, and 23 chitinase proteins from *Arabidopsis*, cucumber, and watermelon, respectively, to examine the evolutionary origin and putative function of the *ClChi* genes (Figure 2). The members of these chitinase were divided into the GH18 and GH19 glycosyl hydrolase families. Based on the phylogeny and sequence homology of *Arabidopsis*, this tree had five clades that were labeled as Class I, Class II, and Class IV that were members of GH19, and Class III and Class V that were members of GH18. As the largest clade, nine chitinase proteins of *Arabidopsis*, eight *ClChi*, and eight *CsChi* proteins were members of Class V. Class II and Class III each contained 15 members. A total of eleven and nine chitinase proteins were members of Class IV and Class I, respectively. Interestingly, in Class I, Class IV, and Class V, cucumber and watermelon had the same chitinase members.

**Figure 3:**
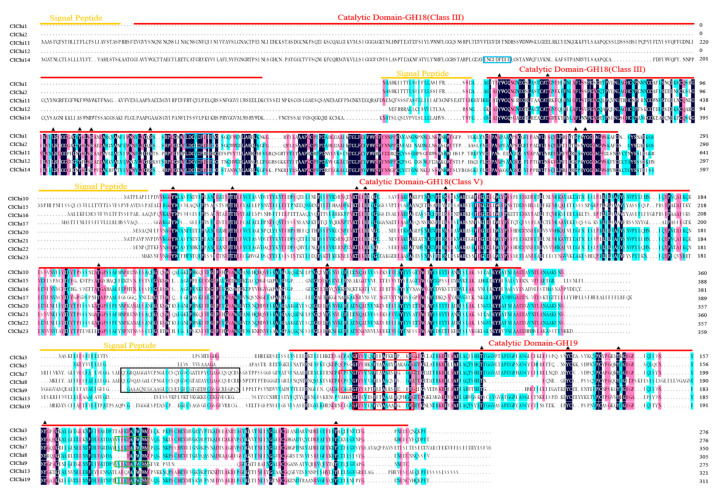
Multiple alignments of the GH18 chitinase Class III and Class V and the GH19 chitinase subfamily of the ClChis sequences. Orange and red lines over sequences indicate signal peptides and their catalytic domains. Blue box: Glycosyl hydrolases family 18 (GH18) active site signature PS01095. Black box: chitin binding domain signature PS00026. Red box: Chitinases family 19 signature 1, PS00773. Green box: Chitinases family 19 signature 2, PS00774. The position of the conserved serine (S), threonine (T), and tyrosine (Y) predicted to be the phosphorylation sites are indicated by the black triangles.

**Figure 4:**
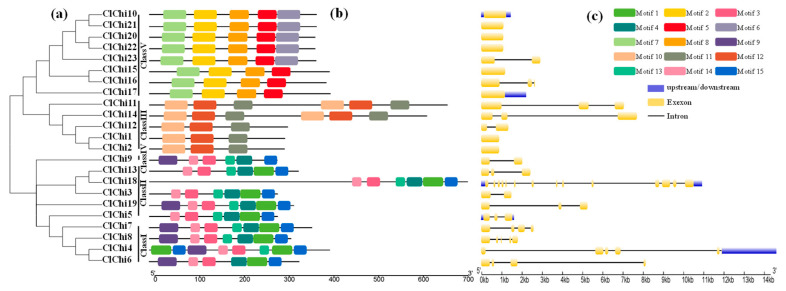
Phylogenetic relationships, conserved motifs, and gene structures of the watermelon chitinases. (**a**) Phylogenetic tree of 23 watermelon chitinase proteins. (**b**) Distribution of the conserved motifs in the watermelon chitinases. (**c**) Gene structure of the predicted *ClChi* genes. Yellow boxes and black lines represent exons and introns, respectively.

Multiple sequence comparisons of the watermelon chitinase amino acids indicated that most of the ClChi sequences contained active sites in their catalytic domain: CHITINASE_18 (PS01095) active site signature, CHITINASE_19_1 (PS00773) and/or CHITINASE_19_2 (PS00774) in the GH18 catalytic domain and GH19 catalytic domain, respectively (Figure 3). The N-terminal of ClChi7, ClChi8, and ClChi9 contained recognition domain signatures (PS00026). In addition, the GH18 domains in Class III and Class V lacked chitin binding domains at their N-terminal. Additionally, the conserved serine, threonine, and tyrosine predicted to be the phosphorylation sites were indicated in the catalytic domains. To identify the conserved motifs of ClChi members, their sequences were analyzed, and the composition of conserved motifs that were consistent with the evolutionary relationships were determined (Figure 4b). A total of 15 conserved motifs were found among the ClChi members. Notably, the members in sister branches or the same group had similar conserved domains. For example, eight ClChis in Class V had the same four conserved domains (motifs 2, 5, 7, and 8), and motifs 10, 11, and 12 were conserved in Class III. In Class I, the motifs 1, 3, 4, 13, 14, and 15 were conserved. Motifs 14, 3, 13, 4, 1, and 15 displayed in the same order were found in the members of GH19; motifs 10, 12, and 11 displayed in the same order were predicted in Class III, and motifs 7, 2, 8, and 5 were displayed in the same order in Class V. In addition, two repeated sequences of motifs (motifs 10, 12, and t11) were found on ClChi11 and ClChi14.

### 2.3. Structural Feature and the Prediction of cis-Acting Regulatory Elements of the ClChi Genes

To study the structural conservation and diversity of the chitinase genes, the exon-intron architecture distribution was characterized with their full length CDS and corresponding genomic DNA sequences based on the phylogenetic relationships (Figure 4c). Eight of thirteen *ClChi* members of the GH18 subfamily had just one exon; *ClChi12* and *ClChi23* had two exons, and *ClChi11*, *ClChi14*, and *ClChi16* had three exons. However, the *ClChi* members of GH19 had at least two exons, and *ClChi18* had the largest number of exons at 14.

**Figure 5:**
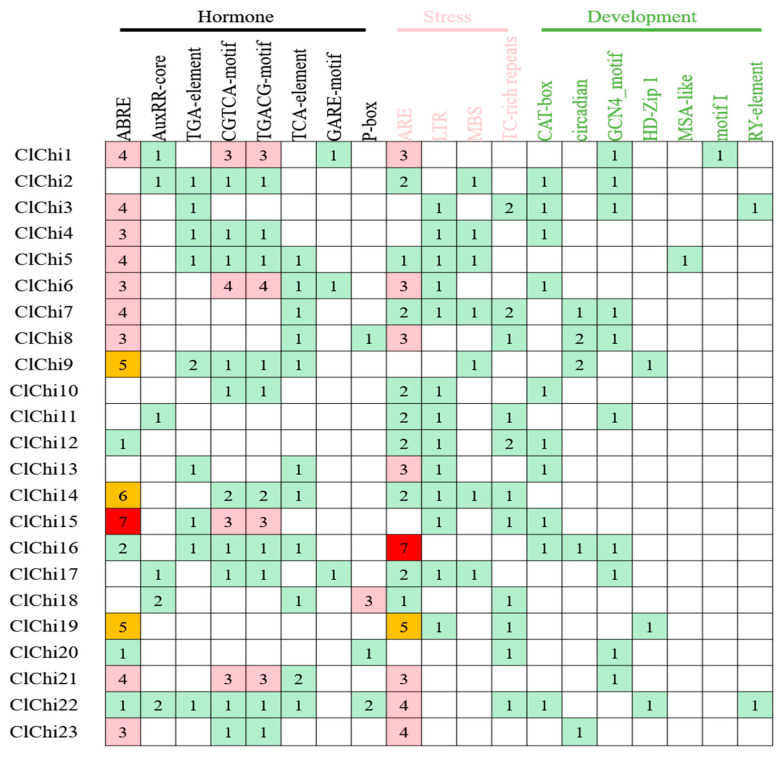
Frequency of the occurrence of *cis*-acting elements upstream of the promoter sequences of chitinases under the functions. Hormone: ABRE, abscisic acid-responsive element; AuxRR-core and TGA-element, auxin-responsive element; TCA-element, salicylic acid-responsive element; CGTCA-motif and TGACG-motif, MeJA-responsive elements; GARE-motif and P-box, gibberellin-responsive elements; Stress: ARE, involved in the anaerobic induction; LTR, low-temperature-responsive element; MBS and TC-rich repeats, involved in defense and stress responsiveness; Development: CAT-box, circadian, GCN4_motif, HD-Zip I, MSA-like, motif I, and RY element, involved in meristem, circadian-controlled differentiation of the palisade mesophyll, expression of the endosperm, differentiation of the palisade mesophyll cells, cell cycle regulation, root-specific regulation, and seed-specific regulation, respectively. MeJA, methyl jasmonate.

The sequences that were found 1.5 kb upstream from the start codon of the *ClChi* gene were submitted to the PlantCARE server to analyze the *cis*-acting elements bound in the promoter. *cis*-acting elements with more than 19 different functions that are involved in phytohormone response and defense and stress responsiveness, as well as the process of plant growth and development, were identified in the promoter region of *ClChis* (Figure 5). In detail, the *cis*-acting elements involved in the phytohormone response included the following: one abscisic acid-responsive element (ABRE), two auxin-responsive elements (AuxRR-core and TGA-element), one SA-responsive element (TCA-element), two MeJA-responsive elements (CGTCA-motif and TGACG-motif), and two gibberellin-responsive elements (GARE-motif and P-box). The predicted stress-related *cis*-acting element included one element involved in anaerobic induction (ARE), one low-temperature-responsive element (LTR), and two elements involved in defense and stress responsiveness (MBS and TC-rich repeats). Moreover, there were seven elements involved in different growth and development processes of the plant, including a CAT-box involved in meristem expression, Circadian-controlled differentiation of the palisade mesophyll, GCN4_motif involved in endosperm expression, HD-Zip I involved in the differentiation of the palisade mesophyll cells, and MSA-like involved in cells cell cycle regulation. Motif I is a root-specific regulatory element and an RY element involved in seed-specific regulation. More than one element belongs to phytohormone response and stress responsiveness, and the regulation of plant development can be found to occur in each promoter sequence, which suggests that the *ClChis* are involved in plant growth and development by responding to various environmental factors.

### 2.4. Profiles of the Expression of the ClChi Genes in Different Tissues

**Figure 6:**
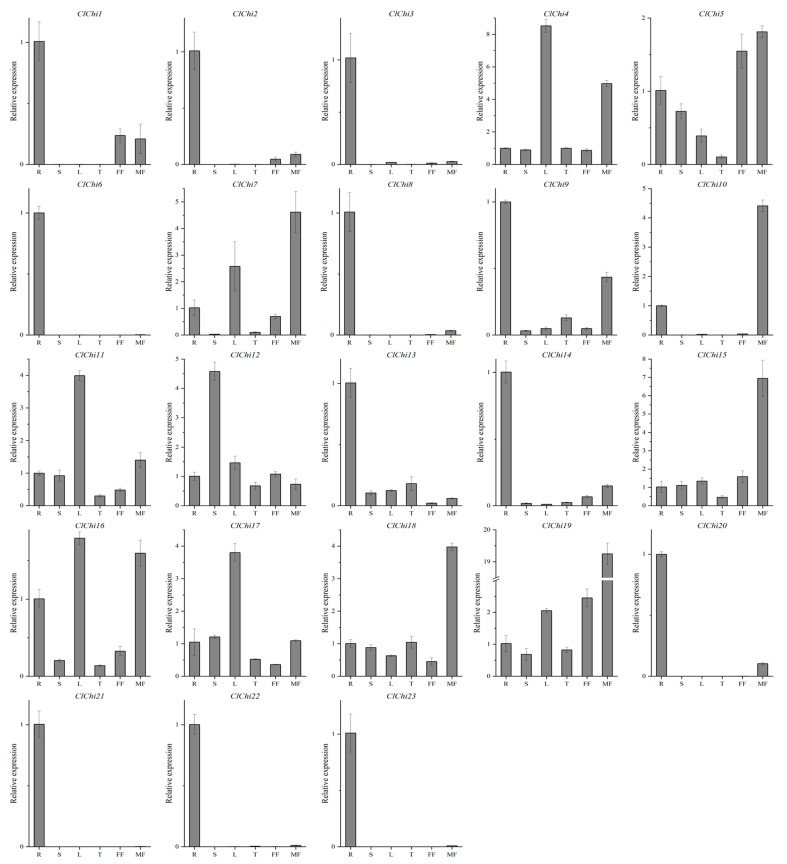
The relative levels of expression of the *ClChi* genes in different tissues. Root (R); Stem (S); Leaf (L); Tendril (T); Female flower (FF); Male flower (MF). All the data points are the means ± SE (*n* = 3).

To investigate the potential functions of the *ClChi* genes in different tissues, the roots, stems, leaves, tendrils, female flowers, and male flowers were collected for real-time quantitative reverse transcription PCR (qRT-PCR) analysis. The levels of expression of the *ClChi* genes can be detected in most selected organs, but their levels of transcription varied (Figure 6). A total of 12 of the 23 *ClChi* members (*ClChi1–3*, *6*, *8*, *9*, *13*, *14*, and *20–23*) were primarily expressed in the roots compared to other organs, while more mRNA accumulated only in the stems in *ClChi12*. *ClChi11* and *17* may play critical roles in leaves owing to the detection of their higher levels of expression in the leaves compared to other organs. There were five members (*ClChi10*, *15*, *18*, and *19*) that were specifically expressed in the male flowers. Furthermore, the abundances of *ClChi4*, *7*, and *16* transcripts were higher in the leaves and male flowers. These results indicate that different *ClChi* gene members are involved in different physiological and developmental processes of watermelon in varying tissues or organs.

### 2.5. Patterns of Expression of the ClChi Genes under Abiotic Stresses

**Figure 7:**
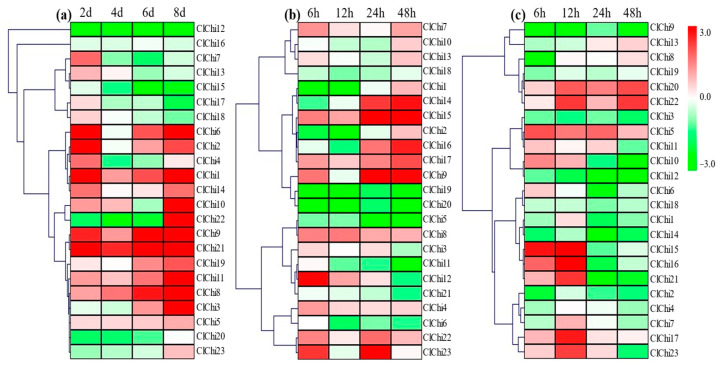
Heatmap of the expression of the *ClChi* genes in ‘M08’ under drought (**a**), salt (**b**), and low-temperature (**c**) stress. Red and green correspond to strong and weak expression of the *ClChi* genes, respectively. The plant samples at 0 dpt and 0 hpt were considered to be controls. dpt, days post-treatment; hpt, hours post-treatment.

The chitinases involved in the responses to abiotic stress of various plants have been reported in previous studies. However, further study is needed to clarify the roles of *ClChis* in resisting external stress in watermelon. With drought treatment, the pattern of expression of the *ClChi* members varied considerably (Figure 7a). Among the 23 *ClChi* genes, the profiles of expression of 16 members (*ClChi1–6*, *8–11*, *14*, and *19–23*) were upregulated with the increase in the time of drought treatment, and three (*ClChi20*, *22*, and *23*) of them were downregulated until 8 days post-treatment (dpt). In contrast, the transcription of four *ClChi* genes (*ClChi7*, *13*, *17*, and *18*) increased slightly at the early stage and then reduced. *ClChi12*, *15*, and *16* were responses to drought with low levels of expression throughout the process of treatment. The patterns of expression of the *ClChi* gene members were studied to determine their responsiveness to low-temperature treatment (Figure 7b). Under cold stress, eleven *ClChi* genes (*ClChi3*, *4*, *7–9*, *12*, *13*, *15*, *17*, *22*, and *23*) were immediately induced to be upregulated 6 h post-treatment (hpt), and six of these eleven members were consistently upregulated throughout the cold treatment (*ClChi4*, *7*, *8*, *15*, *17*, and *22*). Conversely, twelve *ClChi* genes were detected with a low content of mRNA at 6 hpt, and seven of them were downregulated from the beginning to the end of the treatment (*ClChi5*, *6*, *11*, and *18*–*21*). Moreover, high levels of *ClChi14* and *ClChi16* transcripts were detected until 24 and 48 hpt, and *ClChi1*, *ClChi2*, and *ClChi10* were only upregulated 48 hpt. To predict the potential function of the *ClChi* genes in the response of watermelon to osmotic pressure, the patterns of expression of these genes were analyzed under salt stress. As shown in Figure 7c, eleven *ClChi* gene members reduced their levels of expression at the initial stage, and eight members were consistently under-expressed during the treatment (*ClChi2*–*4*, *9*, *12*, *14*, *18*, and *19*). Of the remaining four genes, *ClChi1* and *ClChi7* only had relatively high levels of transcription 12 hpt. *ClChi13* had a higher abundance of RNA transcription 24 and 48 hpt, and *ClChi8* was only transcriptionally upregulated 48 hpt. Except for that, four members (*ClChi5*, *17*, *20*, and *22*) had a higher content of mRNA at all times post-treatment. The significantly upregulated pattern of expression of four *ClChi* genes (*ClChi10*, *15*, *16*, and *21*) were observed 6 and 12 hpt and then decreased. These results of expression under various abiotic stresses suggest that the *ClChi* genes act as important plant regulators.

### 2.6. Patterns of Expression of the ClChi Genes under Hormone Treatments

**Figure 8:**
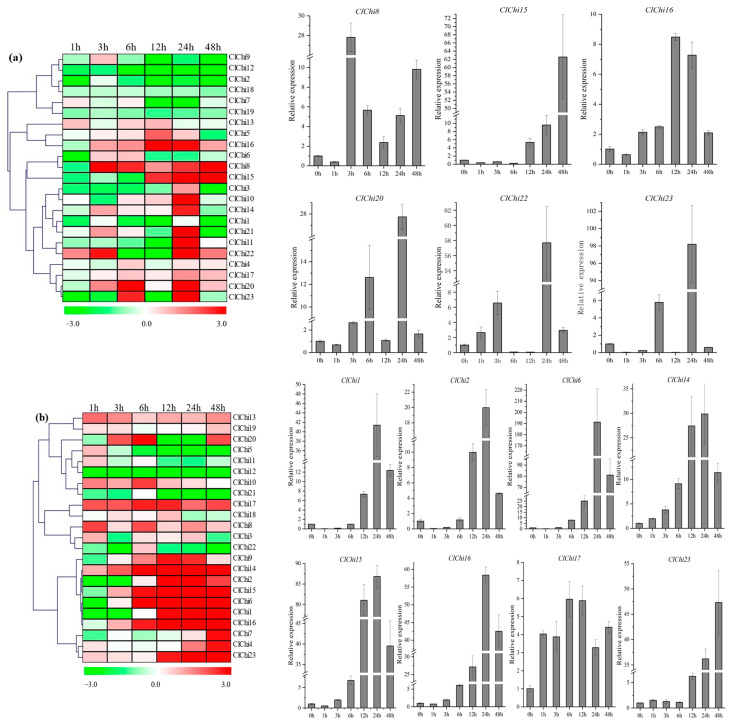

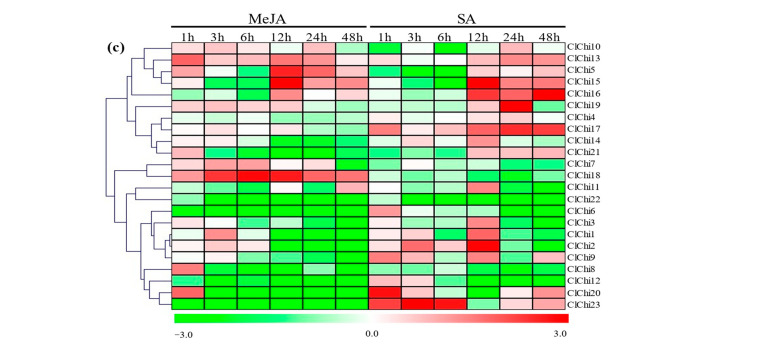
Heatmap of the levels of expression of the *ClChi* genes under ABA, ETH, MeJA, and SA hormone treatments. (**a**) Levels of expression of *ClChis* under ABA stress visualized as a heat map (**Left**). Detailed patterns of expression of the *ClChis* under ABA stress (**Right**). (**b**) Levels of expression of the *ClChis* under ETH stress visualized as a heat map (**Left**). Detailed patterns of expression of *ClChis* under ETH stress (**Right**). (**c**) Levels of expression of the *ClChis* under MeJA and SA stress visualized as a heat map. The plant samples at 0 dpt and 0 hpt were considered to be controls. ABA, abscisic acid; ETH, ethylene; MeJA, methyl jasmonate; SA, salicylic acid.

To explore the mechanism of the response of *ClChi* genes to phytohormones in watermelon, the profiles of expression of the *ClChi* members were studied under four different plant hormones (ABA, MeJA, SA, and ETH). Various patterns of expression of the *ClChi* genes were observed under four treatments. Downregulation of the expression of *ClChi* members was identified 1 htp after ABA treatment, except for *ClChi7*, *13*, and *22* (Figure 8a). However, as the treatment progressed, most of the *ClChi* members accumulated transcripts following induction by ABA. In contrast, the levels of expression of *ClChi1*, *2*, *12*, *18*, and *19* were reduced throughout the whole period of ABA treatment. Remarkably, significantly upregulated levels of expression of six *ClChi* members (*ClChi8*, *15*, *16*, *20*, *22*, and *23*) were observed. Following treatment with ETH, most of the *ClChi* gene members were induced with high levels of transcription, particularly eight members (*ClChi1*, *2*, *6*, *14*-*17*, and *23*) (Figure 8b). However, the levels of expression of five *ClChi* genes (*ClChi5*, *11*, *12*, *21*, and *22*) were downregulated. Three of them (*ClChi5*, *11*, and *22*) were slightly upregulated 1 hpt and/or 6 hpt. Interestingly, the *ClChi* genes exhibited similar or contrasting patterns of expression in response to the stimuli of MeJA and SA (Figure 8c). For example, *ClChi7* and *ClChi18* accumulated more mRNA during MeJA treatment, whereas their expression was downregulated in response to SA treatment. In contrast, the transcription of *ClChi9* and *ClChi23* was inhibited by MeJA and induced by SA. SA seemed to have a greater impact on the levels of transcription of *ClChi1*, *2*, and *17*, despite the fact that they are only marginally upregulated in response to MeJA. Except at 12 hpt and 48 hpt, the levels of expression of *ClChi10* increased during treatment with MeJA. In contrast, it was only highly expressed 24 hpt under treatment with SA. The *ClChi* gene members exhibited a variety of patterns of expression in response to different treatments with plant hormones. This indicated that there are complex regulatory mechanisms that mediate the levels of expression of chitinase genes in plants.

### 2.7. Patterns of Expression of the ClChi Genes in Response to Infection with Fon

**Figure 9:**
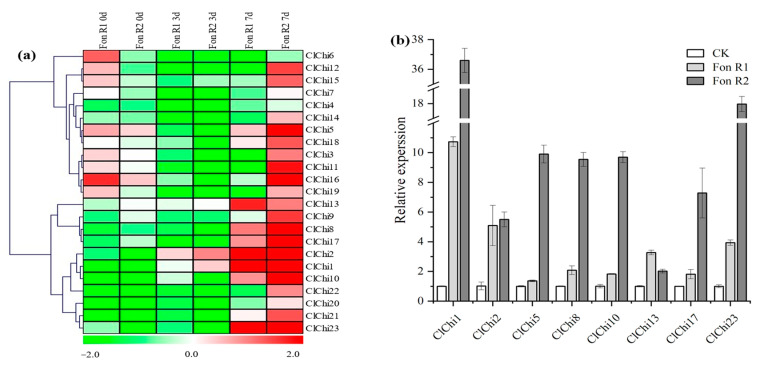
(**a**) Heatmap of the expressed *ClChi* genes in the root tissue of ‘M08’ after infection with the causal agent of Fusarium wilt. The gene clusters were generated using the average linkage clustering method. (**b**) Detailed patterns of expression of *ClChi1*, *2*, *5*, *8*, *10*, *13*, *17*, and *23* infected with *Fon* R1 and *Fon* R2 at 7 dpt. *Fon*, *Fusarium oxysporum* f. sp. *niveum*; R1, race 1; R2, race 2.

To examine the potential roles of the responses of the *ClChi* genes to biotic stress, a cultivated variety of watermelon designated ‘M08’ was infected with *Fusarium oxysporum* f. sp. *niveum* (*Fon*), the causal agent of Fusarium wilt in watermelon. This cultivar is resistant to *Fon* race 1 (R1) and susceptible to *Fon* race 2 (R2). Root tissue samples were independently collected from uninfected and infected plants to explore the patterns of expression of the *ClChi* genes. As shown in Figure 9, the upregulated transcription of nine (*ClChi3*, *5*–7, *11*, *12*, *15*, *16*, and *19*) and two (*ClChi5* and *16*) *ClChi* members were observed 0 dpt of infected R1 and R2, respectively. At 3 dpt, only *ClChi1* and *ClChi2* had accumulated mRNA, while the rest of members were downregulated. The levels of expression of 10 and 21 *ClChi* members (except for *ClChi4* and *6*) increased in the watermelon plants infected with R1 and R2 7 dpt, respectively. At 7 dpt of *Fon* infection, eight *ClChi* gene members (*ClChi1*, *2*, *5*, *8*, *10*, *13*, *17*, and *23*) were significantly upregulated in the treatments of infection with both R1 and R2 (Figure 9b). Thus, the differential expression of the *ClChi* genes observed in response to infection with two different races of *Fon* suggests that these genes could play a vital role in the resistance of watermelon to pathogens.

## 3. Discussion

As a class of proteins with broad-spectrum resistance, chitinases have been implicated in the interactions between plants and microorganisms and/or insects. In addition, chitinases are widely present in prokaryotes and eukaryotes and play roles in regulating their growth and development [10]. Despite a limited description of the systematic examination of the chitinase gene family in certain species, their potential functions in watermelon have not been thoroughly investigated. In this study, a family that consisted of 23 genes that encoded watermelon chitinase was identified using a genome-wide search approach. Their chromosomal locations, collinearity, and *cis*-elements that act on ranges of promoters, gene structures, phylogenetic relationship, and patterns of expression in response to biotic stress, phytohormones, and Fusarium wilt were characterized to elucidate the potential function of the chitinases involved in the response of watermelon to external stress and development.

The *ClChi* genes are unevenly distributed on the watermelon chromosomes (Figure 1). In detail, the chitinase members were primarily concentrated on chromosomes 1, 3, 4, 5, 6, 7, 10, and 11. Moreover, the members of chitinase genes clustered on the same chromosome can be classified into the same class. For example, *ClChi4*, *6*, *7*, and *8* are members of Class I and clustered on chromosome 1. *ClChi11*, *12,* and *13* are clustered on chromosome 5 and classified as Class III. *ClChi15*, *16*, and *17* and *ClChi 20*, *21*, *22*, and *23* are members of Class V and clustered on chromosomes 6 and 11, respectively. The pattern of distribution of the watermelon chitinases might be explained by the tandem duplication events that occurred during evolution, which is similar to those of other species, such as cucumber [6] and cabbage [19]. Furthermore, similar structural features and the composition of motifs have been observed in the members classified into the same class. For example, the conserved motifs 2, 5, 7, and 8 were found on the *ClChi* members in Class V. The intron number and CDS lengths of the gene pairs in the sister branch (*ClChi10* and *ClChi21*, *ClChi20*, and *ClChi22*, *ClChi15* and *ClChi16*) were nearly the same (Figure 4). In addition, the chitinases in classes I and II probably share a more recent common ancestor and together constitute a monophyletic group along with those in Class IV [55]. A similar composition of motifs was observed in the watermelon chitinases of classes I, II, and IV, which suggests that the members of these classes may have similar functions.

In this study, 18 out of the 23 *ClChi* members identified have two or fewer introns (Figure 4c), and most of them were classified into classes III and V. This finding is consistent with the hypothesis that stress-related genes have fewer introns and are rapidly regulated during stress [56]. This is supported by previous studies, including those of chitinase genes in garlic and *Brassica rapa* that were induced in the early stages of infection with *Fusarium proliferatum* and clubroot, respectively [21,57]. A signal peptide located at the N-terminus of most plant chitinases is responsible for their secretion after posttranslational modification [58]. As shown in Table 1, more than half of the ClChi proteins (69.5%) possess N-terminal signal peptides, which suggests that these proteins have potential travel functions. In this study, the catalytic domains were observed in 20 putative ClChi proteins (except for ClChi4, 6, and 18), indicating that these enzymes are functionally conserved with respect to the hydrolysis of chitin (Figure 3). In addition, the phosphorylation sites were found on the conserved catalytic domains of the ClChi proteins. This indicates that the catalytic domains of the ClChi members are likely to be their active regulatory regions and they were regulated by phosphorylation/dephosphorylation, which is controlled by a protein phosphatase/kinase.

Chitinases play a crucial role in plant growth and development, as well as in the interaction between plant/environmental stress. In *Arabidopsis*, *AtCTL1* is a chitinase-like gene that is expressed in all the organs, but it was not induced by stress [59]. *AtCTL1* is involved in the biosynthesis of cell walls and the cellular elongation of roots based on the phenotypes of shorter primary roots and more lateral roots of the mutant under high nitrate treatment [60]. Additionally, aberrant shapes of the cells were observed in the pith of mutant inflorescence stems. Interestingly, the specific expression of the homologs *ClChi3* and *13* in watermelon were detected in the root tissues (Figure 6), which indicate that they have similar functions in root development. The remaining homologs, *ClChi5*, *18*, and 19, are expressed in all the tissues but accumulated more mRNA in the female and/or male flowers. This suggests they may play a crucial role in the floral organs of watermelon. *BC15/OsCTL1* encodes a chitinase-like protein in rice, which is highly expressed in the internodes and nodes. A reduction in the content of cellulose content and mechanical strength phenotype of the *bc15* mutants was observed owing to their thinner sclerenchyma cell walls than those of the wild-type plants [61]. In this study, no particular *ClChi* gene was highly expressed in the stems (except *ClChi12*) or tendrils, which revealed that chitinase may play a relatively inessential role in the development of watermelon stems and tendrils.

Plants often suffer from a variety of external stresses, such as drought, low temperature, and salinization, among others, owing to their immobile characteristics and human activities [62]. Thus, plants have evolved a number of defensive systems to adapt to severe habitats. It has been reported that chitinases play crucial roles in the resistance of plants to external stress. In this study, seedling watermelon plants were treated with drought, salt, low temperatures, and four phytohormones to explore the potential function of chitinase in abiotic stress. The *ClChi* members exhibited varied patterns of expression with different treatments (Figure 7 and Figure 8). For example, *ClChi5* and *11* were induced by drought and salt but not by low temperatures, and greater amounts of the accumulated mRNA of *ClChi8* and *9* were detected in drought, low temperatures, and ETH conditions, but not in salt or MeJA treatments. The level of expression of *ClChi12* was upregulated only in the first 24 h of low-temperature treatment and in the first 3 h of SA treatment, while it was downregulated by treatment with drought, salt, ABA, ETH, and MeJA. The level of expression of *ClChi16* decreased at 6 and 12 htp, followed by an increase in the level of expression at 24 and 48 hpt under low temperatures. This was in contrast to the pattern of expression of this gene under salt, which suggested that it differs in its sensitivity to low temperatures and salt stress. Moreover, *ClChi16* was induced by ABA, ETH, MeJA, and SA. Similarly, the strong accumulation of the *CAChi2* transcripts was observed in the stems of pepper following treatment with salt, drought, and ABA [63]. In sugarcane, the transcription of *ScChi* was significantly induced following treatment with SA, MeJA, ABA, NaCl, polyethylene glycol (PEG), and low temperatures [38]. Although *cis*-acting elements in response to different plant hormones were predicted in the promoter region of members of *ClChi*, patterns of different expression were detected in the *ClChi* genes under identical hormone treatments, which is consistent with the findings in other species [64,65].

The activity of chitinase increases dramatically following plant–pathogen interactions [66,67]. Eight *ClChi* members (*ClChi1*, *2*, *5*, *8*, *10*, *13*, *17*, and *23*) accumulated significant amounts of mRNA in the root tissue infected with R1 and R2 at 7 dpt (Figure 9b). Six of these genes (*ClChi1*, *2*, 8, 13, and 23) were found to be specifically expressed in root tissues, which suggests that they play a crucial role in defense to *Fon*. Alternatively, it was observed that the number of *ClChi* genes and the levels of expression induced by R2 were much higher than those induced by R1. This could possibly be due to the characteristics of ‘M08’ that are resistant to R1 but are susceptible to R2 and the many accumulated R2 spores in the watermelon at the later stage of infection. Moreover, R2 has a stronger pathogenic ability compared to R1. Thus, the watermelon plants have to activate more genes involved in disease resistance to defend against the invasion of exogenous pathogens. Similarly, half of the 28 *CsChi* genes were significantly upregulated with the inoculation of *F. oxysporum* f. sp. cucumerinum (*Foc*) Owen race 3 in a susceptible line of cucumber, while only seven members of *CsChi* were induced in the resistant line [6]. In the resistant cucumber lines, *CsChi23* was induced by infection with *Foc*, and the plant that overexpressed *CsChi23* was resistant to Fusarium wilt and accumulated less fungal biomass, while the silenced plant lacked this resistance [68]. In contrast, the levels of expression of the orthologous chitinase genes *ClChi4* and *ClChi6* were downregulated during the process of infection (Figure 9a). This may be because these two genes were mutated during the gene duplication events, which resulted in a loss of binding/catalytic ability [69]. Despite that, the catalytic domain was observed in the remaining members (*ClChi7* and *ClChi8*) of Class I (Figure 3), which were induced by infection with *Fon*. Their homologous chitinase gene, *TcChi1*, was isolated from cacao fruit based on its pattern of expression following treatment with a fungal elicitor [33]. In addition, the overexpressed plant of *TcChi1* exhibited resistance to the foliar pathogen *Colletotrichum gloeosporioides*, the causal agent of anthracnose.

In conclusion, the results obtained could be used to understand the biological functions of *ClChi* proteins in the development of watermelons and their responses to abiotic/biotic stress. However, the comprehension of the exact roles and mechanisms of action of the genes remains limited. The specific factors that induce the expression of chitinase under abiotic stress and the regulatory mechanism merit further study and elaboration. Thus, further functional investigation of this chitinase gene family is necessary to breed crops to become resistant to biotic and abiotic stress.

## 4. Materials and Methods

### 4.1. Identification and Confirmation of the Domain of ClChis

To identify and characterize the chitinase genes in watermelon, the whole genome sequence and peptide sequence were obtained from the watermelon 97103 genome V2.5 database (http://cucurbitgenomics.org/v2/ftp/genome/watermelon/97103/v2.5/, accessed on 2 May 2023), and the related sequences of the chitinase genes in *Arabidopsis thaliana* (https://www.Arabidopsis.org/, accessed on 2 May 2023) and cucumber (http://cucurbitgenomics.org/organism/20, accessed on 3 May 2023) were obtained as previously described [6,10]. In addition, a BLAST search was performed for the *ClChis* in watermelon genome database (http://cucurbitgenomics.org/v2/organism/16, accessed on 2 May 2023) with the chitinase sequences of *Arabidopsis* as the query. The sequences of candidate *ClChi* were submitted to the NCBI conserved domain database (https://www.ncbi.nlm.nih.gov/cdd, accessed on 14 May 2023), SMART database (http://smart.embl-heidelberg.de, accessed on 14 May 2023), and Pfam database (http://pfam.xfam.org/search/sequence, accessed on 14 May 2023) to identified the conserved signature of glycosyl hydrolase family 18 or 19. The conserved domains were identified by Multiple Em for Motif Elicitation database (https://meme-suite.org/meme/index.html, accessed on 16 May 2023).

### 4.2. Chromosomal Location, Synteny, Gene Structure, Protein Properties, cis-Regulatory Elements, and Phylogenetic Analysis of the ClChis

The chromosomal distributions of the *ClChi* genes were drawn using the online tools of MG2C (http://mg2c.iask.in/mg2c_v2.0/, accessed on 7 June 2023). The relationships between *Arabidopsis*, cucumber, and watermelon were verified and visualized by the Circos tool (http://circos.ca/, accessed on 10 June 2023) for collinearity analysis after the related synteny blocks and duplicated gene pairs were obtained in *Arabidopsis*, cucumber, and watermelon using TBtools (v1.113) software. In addition, the gene sequences and CDSs were submitted to the Gene Structure Display Server (http://gsds.gao-lab.org, accessed on 13 June 2023) to produce the schematic diagram of the pattern of exon/intron distribution. The molecular weights (MWs) and isoelectric points (pIs) of the ClChis were predicted by the Protparam tool (http://expasy.org, accessed on 13 June 2023), and WoLF PSORT (http://www.genscript.com/psort/wolf_psort.html, accessed on 15 June 2023) and SignalP (https://services.healthtech.dtu.dk/services/SignalP-5.0/, accessed on 15 June 2023) were used to predict the subcellular localization and signal peptide, respectively. The *cis*-regulatory elements in the promoter regions of the *ClChi* gene were predicted by the online tools PlantCARE Server (http://bioinformatics.psb.ugent.be/webtools/plantcare/html, accessed on 20 June 2023) with the submitted 1.5 Kb upstream sequences from the starting codon. The phylogenetic relationships of *Arabidopsis*, watermelon, and cucumber were analyzed by the MEGA 7.0.21 program using the neighbor-joining method and the 1000 bootstrap interactions test. Multiple sequence alignments of ClChis and the conserved or similar amino acids were highlighted by using ClustalX2 (v2.1) software, and the conserved phosphorylation sites predicted by NetPhos 2.0 (http://www.cbs.dtu.dk/services/NetPhos, accessed on 22 June 2023). The active site signature and catalytic and chitin binding domain were predicted using the ExPASy prosite (https://prosite.expasy.org/, accessed on 23 June 2023).

### 4.3. Plant Material and Abiotic Stresses Treatments

The cultivated variety of watermelon ‘M08’ was provided by the Cucurbits Germplasm Resource Research Group at the College of Horticulture of Northwest A&F University in Xianyang, China. Germinated ‘M08’ seeds were gently sown into propagation trays in a phytotron with controlled growth conditions as follows: a 28 °C, 14 h light/22 °C, 10 h dark photoperiod, photosynthetic photon flux density (PPFD) of 600 μmol m^−2^ s^−1^, and relative air humidity of 60–80%. The roots, steams, tendrils, leaves, and female flowers’ and male flowers’ organs were sampled independently when the plants were at the flowering period to study the profiles of expression of the *ClChi* genes. Four-week-old seedings were used for the abiotic stresses and phytohormone treatments. In addition, 15-day-old seedlings of ‘M08’ were prepared for inoculation [70], which were resistant to *Fon* race 1 and susceptible to *Fon* race 2.

The watermelon seedlings were treated with drought, salt, and low temperatures to study abiotic stresses. During the process of drought treatment, the leaves of un-watered plant seedlings were collected 0, 2, 4, 6, and 8 dpt. The leaf tissues of seedlings that were watered with 250 mM NaCl were sampled at 0, 6, 12, and 24 hpt. Under low-temperature treatment, partial plant seedlings were incubated at 4 °C with the controlled photoperiod as described, and their leaves were collected 0, 6, 12, and 24 hpt. To study the effects of phytohormones, the plant seedlings were sprayed with 100 μM ABA, 10 mM Ethephon, 1 mM SA, and 100 μM MeJA. The leaves of these four treatments were sampled 0, 1, 3, 6, 12, 24, and 24 hpt. The leaves collected at 0 dpt or 0 hpt were used as controls for both the abiotic stresses and phytohormone treatments.

### 4.4. Preparation and Inoculation of the Fungal Inoculum

The *Fon* races 1 and 2 were cultured on potato dextrose agar (PDA) medium at 28 °C for 2 weeks. Hyphae of the two races were added to 200 mL of Difco™ potato dextrose broth and the spore suspensions were cultured on a rotary shaker for 7 days at 150 rpm and 25 °C conditions. The concentrations of both suspensions of race spores were adjusted to 1 × 10^6^ conidia mL^−1^ with distilled water after the spore suspensions had been filtered through four layers of cheesecloth [71].

The ‘M08’ seedlings were uprooted gently, and the potting soil was washed off. The roots’ tissues were trimmed to create wounds to facilitate fungal invasion. Subsequently, the roots’ tissues were immersed in distilled water and two conidial suspensions of the races for 10 min, respectively. The plants were then replanted in plastic pots (8 cm × 7 cm × 7 cm) filled with a tri-soil mix of perlite: vermiculite: Metromix 360 potting soil (1:1:1, *v*/*v*/*v*). Root samples of the inoculated and control plants were collected 0, 3, and 7 dpt and used for qRT-PCR analyses.

### 4.5. RNA Extraction and qRT-PCR Analysis

The samples in this study from five different plants were pooled at each time-point for each treatment with three biological replicates, and they were frozen in liquid nitrogen and stored at −80 °C for further analysis.

The total RNA from the samples was extracted using an RNASimple Total RNA Kit (TianGen, Beijing, China) and purified using a FastKing RT Kit (TianGen) according to the manufacturer’s instructions. The cDNA was synthesized single-stranded using a FastKing RT Kit (TianGen) and approximately 1 μg of total RNA. In addition, a 20 μL qRT-PCR reaction volume was used, which included 10 μL of SYBR^®^ Green I Master mix (Aidlab, Beijing, China), 0.8 μL of each primer, 2 μL of cDNA template, and 6.4 μL of ddH2O. To amplify their target genes, a specific and efficient primer of internal control (*Claactin-7*) and the *ClChi* genes were used (Table S2). The qRT-PCR process was performed as follows: (1) pre-denaturation at 94 °C for 5 min; and (2) 94 °C for 10 s, 60 °C for 30 s, and 72 °C for 30 s for 40 cycles. The *ClChi* and internal control genes were amplified in triplicate, and the 2^−ΔΔCT^ method was used to calculate the levels of relative gene expression. The levels of expression of all the *ClChi* genes identified were log2 transformed and normalized to generate a heatmap using Mev 4.8.1.

## 5. Conclusions

In this study, a genome-wide analysis of 23 watermelon *ClChi* genes was performed, and the chromosomal location, conserved motifs, gene structures, catalytic domain, phylogeny, and patterns of gene expression were identified based on a bioinformatic analysis and qRT-PCR. These genes were primary expressed in the roots, leaves, and flowers. In addition, multiple patterns of expression were detected under different stress treatments. Additionally, eight *ClChi* genes were identified that played a key role in the resistance of watermelon to Fusarium wilt. The results from this study lay a solid foundation for future research to study the exact roles of chitinase in the development of watermelon and responses to various stresses.

## Figures and Tables

**Table 1:** Characteristics of the *ClChi* genes in watermelon.

Gene Name	Gene ID	Class	Start	End (+/−)	CDS(bp) ^1^	Protein Length (aa)	MW (kDa) ^2^	pI ^3^	*Arabidopsis* Ortholog Locus	E-Value	*Arabidopsis* Locus Description	Signal Peptide ^4^	Subcellular Localization
*ClChi1*	Cla97C01G000780.1	III	525449	526327 (+)	879	293	30.97	9.15	AT5G24090.1	2 × 10^−112^	Chitinase A (class III)	S.P4	Ch
*ClChi2*	Cla97C01G000790.1	III	528158	529033 (−)	876	292	30.72	4.46	AT5G24090.1	9 × 10^−120^	Chitinase A (class III)	S.P	Ch
*ClChi3*	Cla97C01G007600.1	II	7749470	7750940 (+)	831	277	30.79	8.54	AT4G01700.1	8 × 10^−145^	Chitinase family protein	S.P	E
*ClChi4*	Cla97C01G020270.2	I	32998723	33013032 (−)	1170	390	42.02	8.65	AT3G12500.1	3 × 10^−125^	BASIC CHITINASE, PR3	-	N
*ClChi5*	Cla97C01G020300.2	II	33016845	33018436 (+)	831	277	30.61	9.18	AT3G12500.1	2 × 10^−103^	BASIC CHITINASE, PR3	S.P	V
*ClChi6*	Cla97C01G020320.2	I	33013039	33021015 (−)	975	325	34.68	5.34	AT3G12500.1	1 × 10^−135^	BASIC CHITINASE, PR3	S.P	E
*ClChi7*	Cla97C01G020330.1	I	33025312	33027854 (+)	1056	352	37.99	7.39	AT3G12500.1	2 × 10^−140^	BASIC CHITINASE, PR3	S.P	Ch
*ClChi8*	Cla97C01G020340.1	I	33029853	33031634 (+)	918	306	33.2	8.33	AT3G12500.1	1 × 10^−109^	BASIC CHITINASE, PR3	S.P	Ch
*ClChi9*	Cla97C03G057860.1	IV	7034534	7036532 (−)	828	276	29.47	4.68	AT3G54420.1	3 × 10^−141^	CHITINASE CLASS IV	S.P	E
*ClChi10*	Cla97C04G068830.2	V	1947160	1948594 (−)	1083	361	39.82	5.25	AT4G19810.1	6 × 10^−83^	CLASS V CHITINASE	-	Cy
*ClChi11*	Cla97C05G081460.1	III	1167842	1174764 (+)	1929	643	71.58	6.91	AT5G24090.1	7 × 10^−81^	Chitinase A (class III)	S.P	V
*ClChi12*	Cla97C05G081480.1	III	1185858	1187179 (+)	897	299	32.06	8.61	AT5G24090.1	2 × 10^−128^	Chitinase A (class III)	S.P	Ch
*ClChi13*	Cla97C05G088060.1	II	6128296	6130687 (−)	966	322	35.54	6.55	AT3G16920.1	0	Encodes a chitinase-like protein	S.P	Cy
*ClChi14*	Cla97C05G090720.2	III	8775305	8782858 (−)	1797	599	64.59	9.81	AT5G24090.1	2 × 10^−123^	Chitinase A (class III)	S.P	Ch
*ClChi15*	Cla97C06G121340.1	V	23712657	23713823 (+)	1167	389	42.29	5.46	AT4G19810.1	5 × 10^−82^	CLASS V CHITINASE	S.P	E
*ClChi16*	Cla97C06G121350.1	V	23715364	23717968 (−)	1146	382	42.52	8.3	AT4G19810.1	1 × 10^−87^	CLASS V CHITINASE	S.P	Ch
*ClChi17*	Cla97C06G121360.2	V	23724846	23727026 (−)	1173	391	42.74	8.94	AT4G19810.1	9 × 10^−139^	CLASS V CHITINASE	S.P	Ch
*ClChi18*	Cla97C07G143650.2	II	31141290	31151995 (−)	2061	687	75.71	6.41	AT1G05850.1	8 × 10^−160^	CHITINASE-LIKE protein 1	-	E
*ClChi19*	Cla97C10G184910.1	II	425347	430504 (+)	936	312	33.64	5.95	AT3G12500.1	2 × 10^−107^	BASIC CHITINASE, PR3	S.P	Ch
*ClChi20*	Cla97C11G210100.1	V	3572567	3573640 (+)	1074	358	39.72	4.98	AT4G19810.1	4 × 10^−83^	CLASS V CHITINASE	-	Cy
*ClChi21*	Cla97C11G210120.1	V	3586401	3587483 (+)	1083	361	39.75	5.14	AT4G19810.1	1 × 10^−81^	CLASS V CHITINASE	-	Cy
*ClChi22*	Cla97C11G210130.1	V	3591381	3592454 (−)	1074	358	39.79	5.98	AT4G19810.1	1 × 10^−81^	CLASS V CHITINASE	-	Cy
*ClChi23*	Cla97C11G210140.1	V	3598819	3601692 (+)	1080	360	40.67	6.21	AT4G19810.1	8 × 10^−89^	CLASS V CHITINASE	-	Ch

^1^ The length of coding sequences (CDS); ^2^ The molecular weight (MW); ^3^ Theoretical isoelectric point (pI); ^4^ Signal Peptide (S.P); Ch, Chloroplast; Cy, Cytoplasmic; E, Extracellular; N, Nucleus; V, Vacuole.

## Data Availability

The original contributions presented in the study are included in the article/Supplementary Material; further inquiries can be directed to the corresponding author/s.

## Supplementary Materials

The supporting information can be downloaded at: https://www.mdpi.com/article/10.3390/ijms25010638/s1.

## Abbreviations

SAR	systemic acquired resistance
HR	hypersensitive response
ETI	Effector-triggered immunity
PAMP	plant pathogens-associated molecular pattern
PTI	PAMP-triggered immunity
RP proteins	pathogenesis-related proteins
ABA	abscisic acid
ETH	ethylene
MeJA	methyl jasmonate
SA	salicylic acid
htp	hour post treatment
dtp	day post treatment
